# Bridging Exercise Science, Cognitive Psychology, and Medical Practice: Is “Cognitive Fatigue” a Remake of “The Emperor’s New Clothes”?

**DOI:** 10.3389/fpsyg.2018.01246

**Published:** 2018-09-10

**Authors:** Nathalie Pattyn, Jeroen Van Cutsem, Emilie Dessy, Olivier Mairesse

**Affiliations:** ^1^VIPER Research Unit, Royal Military Academy, Brussels, Belgium; ^2^Department of Experimental and Applied Psychology, Vrije Universiteit Brussel, Brussels, Belgium; ^3^Human Physiology Research Group, Vrije Universiteit Brussel, Brussels, Belgium; ^4^Endurance Research Group, University of Kent, Chatham, United Kingdom; ^5^Sleep Unit, CHU Brugmann, Brussels, Belgium

**Keywords:** cognitive fatigue, exercise tolerance, fatigue, sleep disorders, chronic fatigue, effortful control, performance

## Abstract

Fatigue is such a multifaceted construct it has sprouted specific research fields and experts in domains as different as exercise physiology, cognitive psychology, human factors and engineering, and medical practice. It lacks a consensus definition: it is an experimental concept, a symptom, a risk, a cause (e.g., of performance decrement) and a consequence (e.g., of sleep deprivation). This fragmentation of knowledge leads to slower dissemination of novel insights, and thus to a poorer research. Indeed, what may seem as a novel result in one field, may very well be old news in another, hence leading to this “innovation” being a scientific equivalent to the emperor’s new clothes. The current paper aims to describe the common denominator in the different areas of expertise where fatigue is investigated. Indeed, rather than focusing on the differences in semantics and conceptualization, we hope that identifying common concepts may be inductive of easier multidisciplinary research. Considering the vastness of fatigue research in all areas identified as relevant-cognitive science, exercise physiology, and medical practice, this analysis has not the ambition to be an exhaustive review in all domains. We have reviewed the fatigue concepts and research in these areas and report the ones that are used to describe the proposed common model to be further investigated. The most promising common feature to cognitive science, exercise physiology and clinical practice is the notion of “perceived effort.” This allows to account for interindividual differences, as well as for the situational variations in fatigue. It is applicable to both mental and physical constructs. It integrates motivational and emotional dimensions. It overcomes current polemics in various research fields, and it does not draw on any semantic ambiguity. We thus suggest a new model of fatigue and performance, whether this performance is mental or physical; and whether it is in a clinical range or relates to optimal functioning.

## Introduction

Fatigue is typically defined as “extreme and persistent tiredness, weakness or exhaustion-mental, physical or both" (Dittner et al., 2004). Though a vitally important feature in various conditions, fatigue is a very complex and multidimensional construct, studied in a wide variety of research domains. In biological psychology, fatigue has often been investigated within the context of sleep-wake regulation, where studies traditionally targeted central processes, such as subjective perception, well-being, cognitive performance and their underlying neurophysiology. In exercise physiology, on the other hand, fatigue has traditionally been defined on a peripheral level, as “an acute impairment of exercise performance that leads to an inability to produce maximal force output, possibly due to metabolite accumulation or substrate depletion" (Gibson and Noakes, 2004). In ergonomy and human factors, fatigue has been a focus of research ever since the industrial revolution. As Rabinbach (1992) points out, the medical world acknowledged it as early as 1870, when it was formally recognized as a disease of overwork (not too far away from our current concepts of burn-out). Overall, it was then considered as a sign that the human organism has a limited capacity to respond to the demands of modern working life. Di Giulio et al. (2006); Di Giulio (2011) summarizes how the psychophysiological investigation of fatigue finds its roots in nineteenth century evolutionary biology. Indeed, the Italian physiologist Angelo Mosso first studied migratory birds and military pigeons, to investigate their tiredness after hours of flight, as well as the decrease of their stamina with aging. In his book “Fatigue" (1915), which was first published in Italian in 1891, he aimed to combine scientific findings from diverse areas, as well as cultural, social, political, and pedagogic ideas. As such, the first holistic investigation of fatigue was a fact, which he also aimed at explaining physical exhaustion and neurasthenia of workers in this industrial revolution era. Nowadays, even though there is still no scientific consensus on diagnostic criteria and construct validity regarding burn-out (e.g., Bianchi et al., 2015), these seemingly simplistic descriptions from over a century ago still seems to hold true at some level. What is worse, is that we seem to have overlooked significant seminal work from the early days, to reach very similar conclusions as if these were new insights.

The notion of different components of fatigue is well-accepted in the literature. Physical fatigue is dependent of factors such as the type, magnitude and intensity of physical labor and effort as well as neuromuscular characteristics, metabolite storage, buffering capacity, etc. (Bogdanis, 2012). Mental fatigue on the other hand can be conceptualized as an outcome of incremented cognitive load due to constrained time to process perpetual cognitive demands amongst others, independently occurring from sleepiness (see Neu et al., 2010a; Borragán et al., 2016). Fatigue related to sleep deprivation has been described in terms of vigilance decrement and propensity to sleep (Lim and Dinges, 2008), and is thus often confused with sleepiness. Fatigue also has a physiological counterpart, like in acute and very energy demanding activities, but in contrast to sleepiness, it may resolve with rest and does not require sleep to recover from. Despite the relevance of fatigue in various domains, a “gold standard” available for the measurement of fatigue is still missing. Fatigue is quantified through its multiple effects, with different focuses in the different areas of expertise. Fatigue is thus a multidimensional construct, investigated through approaches that depend on the main interest of the research team, hence with a limited focus.

However, in the development of increasingly multidisciplinary research during the past decades, concepts have started to permeate across traditional study field boundaries. Sports science has seen a booming research in what is now called “mental fatigue” (Gibson and Noakes, 2004; Marcora, 2008; Marcora et al., 2009), notwithstanding the fact that this was also Mosso’s conclusion in the late nineteenth century (Mosso, 1915, in Di Giulio, 2011). In cognitive neuroscience, we have seen a shift form the more traditional attention and vigilance research (Mackworth, 1948; Caldwell, 1995; Dinges et al., 1997), with roots in occupational psychology and sleep research, to a field where mental fatigue is investigated related to the constructs of resource depletion, boredom, and motivation (e.g., Pattyn et al., 2008; Gergelyfi et al., 2015). In the clinical field, ranging from oncology to sleep medicine, neurology, and probably most importantly general practice, practitioners consider the lack of a clear conceptualisation of fatigue for diagnostic and treatment purposes to be a major clinical issue (e.g., Guilleminault and Brooks, 2001; Neu et al., 2010a).

The cross-fertilization between fundamental cognitive science, applied occupational psychology, exercise science, and the clinical field is a major progress to shed new light on the protean construct of fatigue. However, to avoid one-eyed kings being partial experts in the land of the blind, we currently advocate a common denominator across areas of expertise for the investigation of fatigue. Hence the current paper. Beyond the apparent provocative title, we wish to advocate for a broader knowledge across disciplines of related investigations focussing on fatigue. Therefore, we present a new model to be used as an investigative framework, merging insights from psychology and physiology; from fundamental research and applied findings.

## Fatigue as A Psychological Construct

Twenty years ago, Baumeister et al. (1998) made a seminal contribution to the study of fatigue: the description of ego depletion. Their core idea was that conscious acts of self-control, defined more broadly as volitional action, draw on some sort of limited resource, meaning that even seemingly different unrelated actions share a common resource pool, and thus influence one another. We will elaborate in the next paragraph about the consequences of integrating these insights in exercise physiology.

However, one aspect of the ego depletion research that is one of the core issues of the investigation of fatigue, is related to the notion that fatigue is caused by the consumption of a limited resource, seen as attention in vigilance research, or metabolic fuel in exercise science, or time spent awake reflected in homeostatic sleep drive in sleep research. More generally, one can argue that this fits within “behavioral energetics,” as has traditionally been used in psychophysiology (Berntson et al., 1993), more specifically related to autonomic nervous system physiology (Pattyn, 2009). A full historical account of the energetical concept of “drive” in psychology is outside the scope of the present paper, but it is precisely because of the importance of such account that the energy question is at the core of the present ambiguities in the description of fatigue. As pointed out by Hockey (2013), the assumption that fatigue is directly caused by a physical loss of energy is probably the most serious failure of traditional fatigue theory. Whereas everybody can intuitively relate to the vocabulary of fatigue regarding lacking energy, it is a gross scientific mistake to extend this experience to scientific evidence. Hockey (2013) identifies this shift from the metaphorical to the factual based on account Lakoff and Johnson’s (1980, in Hockey, 2013) that poorly understood experiences become prime targets for metaphor, which has been the case for fatigue for more than a century. And these metaphors, in return, shape our understanding of the meaning of the construct, effectively crafting a kind of self-fulfilling prophecy in the definition of fatigue.

And yet the energy-depletion account is still a mainstream view in fatigue research, and it is not difficult to see why. For example, the role of glucose as the substrate for either cognitive performance (e.g., Fairclough and Houston, 2004) or for self-control has been investigated by researchers. Glucose (i.a., a sugar) is an essential fuel for the human organism. Multiple experiments have linked self-control depletion to a decreased blood glucose concentration and suggested that self-control depletion could be counteracted by consuming glucose (Gailliot et al., 2007). However, some of these findings were later questioned (Kurzban, 2010). Recent experiments have demonstrated that resource depletion effects can be turned around by mouth rinsing sweet beverages (Molden et al., 2012; Sanders et al., 2012; Hagger and Chatzisarantis, 2013) which can have rewarding properties (Frank et al., 2008; Van Cutsem et al., 2017a). The question yet remains what neural mechanisms may underlie self-control failure. Based on cognitive models of mental control it is suggested that a conflict-monitoring/error-detection system identifies discrepancies between intended goals and actual behaviors (Yeung, 2013), and it is precisely within this system that we might locate the origin of fatigue (Inzlicht and Gutsell, 2007). Error-related negativity (ERN) signals are a waveform of event-related brain potentials, these signals appear to be produced in the anterior cingulate cortex and are associated with committing errors in multiple psychological tasks (Holroyd and Coles, 2002). Using electroencephalography (EEG), Inzlicht and Gutsell (2007) demonstrated that ERN signals were weaker in individuals who had completed an emotion-suppression task compared to individuals who did not complete this task. These findings indicate that neural mechanisms responsible for conflict monitoring can be weakened after having exerted/depleted self-control in a previous task. However, a major caveat in this line of research needs to be identified, and it is that most of ego depletion studies have been carried out in convenience samples such as university students. This of course, raises concerns about the generalizability of the results. The role of age in the effects of ego depletion is rather unclear, however, younger people seem to be more susceptible to these negative effects of ego depletion. Dahm et al. (2011) reported that people over the age of 40 do not become as ego depleted following a typical depletion manipulation as younger university students. This difference in ego depletion between both age categories could be explained, in part, by the fact that the development of the areas of the brain involved in self-control continues until the mid-20 s.

Regarding the roots of the description of fatigue in cognitive science, it can be traced to a very practical question from the work floor. During WWII, the Royal Air Force tasked Norman Mackworth to determine the optimal task duration for radar operators, and Mackworth thus set out to investigate the deterioration of perceptual efficiency due to time-on-task, hence laying the foundations of vigilance research as we have known it for about three quarters of a century now, and in the process defining vigilance as “a psychological readiness to perceive and respond” (Mackworth, 1948). Nowadays, it is conceived as the ability to maintain the focus of cognitive activity on a given stimulation source or task (Eysenck, 1982), and is termed alternatively sustained attention or vigilance.

The cognitive understanding of the cause(s) of the vigilance decrement brings us back to the energy issue, i.e., the description of fatigue caused by a depletion of energetical resources. For the last 20 years, researchers have advocated back and forth between two main accounts for the vigilance decrement. Some authors (e.g., Stuss et al., 1995; Robertson et al., 1997; Manly et al., 1999; Pattyn et al., 2008; Ralph et al., 2017) state that attentional withdrawal of the supervisory attentional system, due to underarousal caused by the insufficient workload inherent to typical vigilance tasks, triggers the vigilance decrement. Several sources of experimental results supporting this hypothesis can be identified. To name a few: “the fact that a correlation was observed between task-irrelevant mental activities and attentional lapses during a vigilance task” (Smallwood et al., 2004); or “the findings showing the highest performance decrements in sustained attention to be associated with lower cortical tonic activation and lower phasic ERP responses” (Dockree et al., 2007); or results showing a deactivation (i.e., a relaxation) of the autonomic nervous system related to a decrement of cognitive performance over time-on-task (Pattyn et al., 2008). Other authors (e.g., Temple et al., 2000; Grier et al., 2003; Helton et al., 2005; Gergelyfi et al., 2015) define the vigilance decrement as the result of a reduced attentional capacity over time-on-task. In other words, the impossibility to sustain the effort, due to the too high mental workload, as corroborated by subjective evaluations by participants. According to this description, performance failure in vigilance because of a depletion in information-processing resources reflects limitations in effortful attention (Grier et al., 2003; Helton et al., 2005; Gergelyfi et al., 2015) observed in conjunction with high ratings on several workload indices can be summarized as “overload.” This could be referred to as the boredom versus cognitive fatigue hypothesis. Hence boredom could be “underload,” from a resource-demands point of view, and cognitive fatigue as an overload. This apparent paradox between the “underload” hypothesis (i.e., the subjective experience of participants indicates that the vigilance decrements are associated with mind-wandering), and the “overload” hypothesis (i.e., the vigilance decrements is associated with high evaluations of workload), is actually resolved when considering Hancock and Desmond’s (2001) distinction of active and passive fatigue. Active fatigue is caused by prolonged, task-related perceptual-motor adjustment. Hence active fatigue reflects the more overload, usually termed “cognitive fatigue” account. Passive fatigue is caused by a prolonged, boring, monotonous task over a prolonged time, thus reflecting the underload or boredom account. Both are termed fatigue by the authors, because of their impact on performance. However, both are caused by very different mechanisms. And, both relate very differently to the issue of energy depletion. However, they mutually relate to the concept of an optimal level of stimulation for performance, with both ends of the spectrum, either too high or too low, leading to decrements. This could be compared to the Yerkes and Dodson (1908) law, where an optimal level of arousal is described for optimal performance, with both underarousal and overarousal being detrimental. In humans, this has been demonstrated by a range of experiments, investigating both the high end of the spectrum (e.g., Pattyn et al., 2014) and the low end (e.g., Pattyn et al., 2008); and even the matching of task difficulty to the arousal of the participant (e.g., Freeman et al., 2004), which thus suggests the existence of an optimum of workload.

This notion of an optimum of workload depending on the subject’s state would account for the importance of interindividual and even intra-individual differences in vigilance research (Borragán et al., 2017). It is precisely this notion of matching between a participant’s state and task demands that allows for an elegant escape from the rigidity of the energy and resource depletion metaphors. Indeed, similarly to the transactional model of stress (Folkman and Lazarus, 1985), the notion of appraisal and matching are included in several conceptual descriptions of fatigue so far. Matthews (2011) for example, defines cognitive fatigue as the result of an individual’s evaluation of task demands and not as high workload *per se*. Hockey (1983, 2013) describes stress and control as mediators of fatigue effects, effectively introducing the perception of effort as the crucial determinant of the occurrence of fatigue. Boksem and Tops (2008) describe motivation as the main precursor for cognitive fatigue, hence effectively introducing a state variable in the concept as well. Richter et al. (2016) provide a link between effort and motivation, by reviewing the application of the “Motivational Intensity Theory” building on work from Brehm and Self (1989) and Wright and Brehm (1989) to the concept of effort. Richter et al. (2016) emphasize how this “Motivational Intensity Theory” was not originally developed to predict effort, but that subsequent work, mainly on the psychophysiological investigation of the theory’s predictions regarding motivation, effort mobilization and task difficulty, led to a conceptualization of effort. Borragán et al. (2017) base their description on the conceptual framework of the “Time Based Resource Sharing model” from Barrouillet et al. (2004), to demonstrate variations in fatigue independent from any other variables than time pressure, hence demonstrating a state-like variation and advocating for a dynamic concept of workload.

## Fatigue in Exercise Science

Within the domain of exercise physiology, fatigue is mainly investigated because of its consequence, being exercise limitation. Exercise-induced fatigue (i.e., the inability to continue a given exercise) is often associated with peripheral and central factors, accordingly the terms peripheral fatigue and central fatigue came in use. Peripheral fatigue is usually described as an impairment located in the muscle and characterized by a metabolic end point, while central fatigue is defined as a failure of the central nervous system to adequately drive the muscle (Meeusen and Roelands, 2018). Regarding peripheral fatigue, mechanisms that are proposed to play a role are for example impaired calcium release from the sarcoplasmic reticulum (Allen et al., 2008) and disturbed muscle ionic homeostasis (i.e., intracellular-interstitial perturbations in K+ and Na+ concentrations) (McKenna and Hargreaves, 2008). Based on the concept of a critical threshold of peripheral fatigue, the role of neural feedback mechanisms related to the occurrence of, for example, impaired calcium release, have been extensively investigated in exercise physiology (McKenna and Hargreaves, 2008). Within this concept it is hypothesized that during exercise, afferent feedback related to this fatigue is provided to various spinal and supraspinal centers by group III and IV fibers (Craig, 2002), and subsequently limits exercise performance. Given the existence of such sensory system, it seems logical these afferent stimuli play a role in the occurrence of exercise-induced fatigue. However, these are insufficient to explain every aspect of exercise-induced fatigue. Marcora and Staiano (2010) for example, demonstrated that participants were able to produce 731W in a maximal voluntary cycling power test immediately after having performed a time-to-exhaustion cycling test at 80% of peak aerobic power. This shows that participants were able to produce a significantly higher amount of power immediately after reaching their “critical” threshold of peripheral fatigue in the time-to-exhaustion test, and thus suggests that the concept of a critical threshold of peripheral fatigue is not generalizable across different exercise modalities.

In contrast to peripheral fatigue, central fatigue refers to fatigue that originates in the central nervous system, spinal and/or supraspinal (i.e., the brain). Operationally, it is often defined as an exercise-induced decrease in maximal voluntary activation level (Pageaux et al., 2015). A mechanism that has been proposed to play a role within this kind of fatigue is for example challenged oxygenation of the brain during exercise (Secher et al., 2008), but also neurochemical and thermodynamic changes of the cerebral homeostasis have been proposed to lead to central fatigue (Kayser, 2003; Nybo and Secher, 2004; Klass et al., 2016). However, similarly to the proposed peripheral mechanisms of fatigue, the reductionistic pitfall to link these central mechanisms with the occurrence of exercise-induced fatigue based on the concept of a critical threshold, has to be avoided, i.e., a linear model in which increases or decreases in brain neurotransmitter concentrations cause fatigue (Gibson and Noakes, 2004).

Based on the multiple factors affecting the occurrence of peripheral and/or central fatigue, several models attempting to summarize the determinants of fatigue have been developed. Abbiss and Laursen (2005) summarized them and highlighted for example the cardiovascular model, energy depletion model and the thermoregulatory model. However, these authors acknowledged the reductionist nature of these models, which are mostly linear and sequential, based on the previously mentioned concept of a critical threshold. This is of course an oversimplification as fatigue is a complex phenomenon that is affected by cardiovascular, respiratory, metabolic and neuromuscular factors and their interactions. Moreover, besides the different physiological processes and their interactions, fatigue-associated factors determined in other research areas than exercise psychology, such as cognitive neuroscience, genetics, and other scientific disciplines must not be ignored in an attempt to understand the exercise-induced fatigue. The importance of this holistic approach to exercise-induced fatigue was already demonstrated in 1891, when Angelo Mosso reported in his seminal book on fatigue that muscle endurance was reduced in two fellow professors of physiology after long lectures and verbal examinations. This interaction between a cognitive task and physical performance already indicated that understanding exercise-induced fatigue would take more than a critical threshold of peripheral fatigue. Mosso was the first to acknowledge the mutual relation between physical and mental performance, stating that “fatigue of the brain decreases muscle strength”; and that muscle fatigue caused an inability to concentrate (Mosso, in Di Giulio, 2011). Gibson and Noakes (2004) made an early attempt to provide a complex, non-linear, dynamic model in which physiological systems interact to regulate activity. The central governor model states that “the brain performs subconscious calculations of the metabolic cost required to complete a given exercise task, and then computes how this will be influenced by the prevailing environmental conditions and the current physical state. This allows the selection of an optimum pacing strategy that will allow completion of the task in the most efficient way while maintaining internal homeostasis and a metabolic and physiological reserve capacity” (Gibson and Noakes, 2004). Despite the usefulness of this model to emphasize the important role of the brain in the regulation of exercise performance and shift away the attention of the concept of a critical threshold of peripheral fatigue, it has been questioned multiple times (Inzlicht and Marcora, 2016). Marcora (2008), for example, pointed out that the model did not adequately consider the prominent role of motivation in exercise performance. To address this issue, he presented the psychobiological model. This model is based on the motivational intensity theory (Wright, 1996) and postulates that exercise-induced fatigue occurs (A) when the effort required by the exercise task is equal to the maximum effort the subject is willing to exert to succeed in the task; or (B), when the subject believes to have exerted a true maximal effort and continuation of exercise is perceived as impossible. According to Marcora (2008), this psychobiological model based on effort-related decision-making may provide a unifying theory of exercise tolerance and suggest that exercise tolerance in highly motivated subjects is ultimately limited by perception of effort. Again, we see that the answer to a current controversy may lie in a transactional explanation of fatigue.

Subsequently, studies on the neurophysiological basis (i.e., based on afferent and/or efferent signals; see Smirmaul et al., 2013) of the perception of effort are emerging within exercise-physiology research (de Morree et al., 2012, 2014; Zénon et al., 2015; Sharples et al., 2016). Besides these novel insights following the publication of the central governor model, the model itself was also updated in order to address the raised concerns. In its most recent update, this model progressed toward “a three-dimensional framework of centrally regulated and goal-directed exercise behavior, which differentiates between sensory, affective, and cognitive processes shaping the perceptual milieu during exercise” (Venhorst et al., 2017).

In the framework of this increased focus on perception of effort, and thus the importance of cognitive mechanisms in exercise tolerance, cognitive fatigue, mental fatigue, and ego depletion have recently known a boost in occurrence in sport science literature (Bray et al., 2008; Marcora et al., 2009; Neyroud et al., 2016; Van Cutsem et al., 2017b). Based on the seminal work form Marcora et al. (2009), these mainly stem from the observations that manipulations inducing mental fatigue (be it cognitive, emotional, active, or passive) impair sport performance (Marcora et al., 2009; Neyroud et al., 2016; Van Cutsem et al., 2017b). Depending on the mechanism that is hypothesized to mediate the occurrence of fatigue, a specific manipulation is chosen, and the fatigue is subsequently termed cognitive fatigue, mental fatigue, or ego depletion/self-regulatory strength depletion. These various kinds of fatigue all refer to the psychophysiological consequence of a psychological intervention. Hence the need for a common denominator, to avoid further confusion. Indeed, reports already show discrepancies, for example in the length of the cognitive activity that is used to induce a particular kind of fatigue and the mechanism that is speculated to trigger this fatigue. In the ego depletion research, tasks as short as 4 min have been argued to deplete self-control and subsequently impair performance (Neyroud et al., 2016). Regarding mental fatigue, Van Cutsem et al. (2017b) argued in their systematic review that cognitive tasks have to be prolonged for at least 30 min in order to be able to result in that kind of fatigue. The rationale behind their choice of terminology was based on the reality of the field (most studies in exercise science investigating physical performance after prolonged cognitive activity termed this kind of fatigue as “mental fatigue” (Bray et al., 2008; Brownsberger et al., 2013; MacMahon et al., 2014; Pageaux et al., 2015) and because “mental” suggests a possible role for emotion and motivation rather than just cognition. On the other hand, Ackerman and Kanfer (2009) and MacMahon et al. (2014) have stated that, considering the typical tasks used to induce fatigue, the term “cognitive fatigue” is more appropriate.

This patchy terminology has created a semantic ambiguity in the field. Indeed, all three terms of fatigue have created a specific research area and there is very little to no communication between these. Within the ego depletion literature, the anterior cingulate cortex (ACC) area of the prefrontal cortex is frequently emphasized to play a major in controlling the effects of ego depletion on physical performance. In order to substantiate this, Bray et al. (2012) for example point out that several studies show increased activation of the ACC during performance of a Stroop task as well as during prolonged submaximal handgrip squeezing. ACC activation during effortful or centrally fatiguing tasks could subsequently deplete blood glucose concentrations and as such increases in cerebral metabolism linked to ACC activation may rapidly consume fuel stores required for maximal effort and account for the effects of ego depletion on physical performance (Bray et al., 2012). This might very well overlook the role of the ACC in the regulation of physical activation through the Central Autonomic Network (e.g., Critchley et al., 2003; Matthews et al., 2004), hereby once more underscoring the pitfalls of reductionism in the study of psychophysiological constructs. The recent evolution of the cognitive fatigue and mental fatigue literature shows that the search for a possible physiological mechanism behind the spill-over effects of cognitive/mental fatigue on physical performance is ongoing. Pageaux et al. (2014) speculated that adenosine accumulation in the anterior cingulate cortex and the pre-supplementary motor area due to a mentally fatiguing task, could partly explain the higher than normal perceived exertion during an endurance exercise in a mentally fatigued state. However, this speculation has, up to date, not yet been tested experimentally. In addition, Van Cutsem et al. (2017b) indicated that other possible neurotransmitters that could play a role in the occurrence and the effects of mental fatigue must not be overlooked. Independently from the mechanism behind the spill-over effects of ego depletion, cognitive or mental fatigue on physical performance, the important take-home message here is that “fatigue stemming from physical and mental exertion may not be separate systems, with the latter co-opting the pre-existing neural machinery of the former” (Evans et al., 2015; Inzlicht and Marcora, 2016).

## Fatigue as A Clinical Condition

Individuals presenting with various chronic conditions often complain about symptoms related to pain, fatigue, and sleep (Penner and Calabrese, 2010). Nearly half of all cancer patients; four out of five patients with rheumatic disease and fibromyalgia syndrome; and up to nine out of ten patients with multiple sclerosis complain about debilitating fatigue (Wolfe et al., 1996; Trojan et al., 2007; Singer et al., 2011). Fatigue as a core symptom is reported to significantly affecting quality of life, with severe health-related and economic consequences (Reeves et al., 2007; Penner and Calabrese, 2010). Furthermore, these patients often suffer from the multifactorial character of their condition: both medical doctors and psychologists feel ill-equipped to deal with the diagnosis and treatment of fatigue, for each feels he/she lacks a part of the expertise.

In clinical practice, fatigue is labeled as a complaint in which sustaining motor or noetic effort levels gets harder in acute and demanding tasks (Shen et al., 2006; Neu et al., 2010b). It is often confused with sleepiness, both by patients and clinicians alike, due to semiologic and semantic similarities (Neu et al., 2010b). However, from a pathophysiological point of view, both symptoms and associated clinical conditions largely differ. Both also express different needs: fatigue needs essentially rest to recover from and sleepiness specifically needs sleep to alleviate it (Mairesse and Neu, 2016). In pathological conditions, excessive daytime sleepiness presents as continuously high levels of sleep pressure and increased sleep propensity, modulated mainly by a sleep-dependent homeostatic process and a sleep-independent circadian oscillator (Neu et al., 2010b; Mairesse et al., 2014). Conversely, physiological fatigue is often considered as acute in nature and pathological fatigue, not alleviated by rest and exacerbated by cognitive or physical tasks, as chronic (Hossain et al., 2003; Neu et al., 2010b). Chronic fatigue is rather related to systemic conditions (e.g., inflammatory processes, immune disorders, cancer, major depression), to insomnia disorder (a primary sleep disorder associated with a hyper-arousal condition and largely without excessive daytime sleepiness) and it is the core symptom of chronic fatigue syndrome (CFS) (Shen et al., 2006; Neu et al., 2010b). Chronic fatigue in CFS patients without any primary sleep disorder and without excessive daytime sleepiness has been associated to several potential impairments of, or complaints about sleep (Neu et al., 2007, 2008; Mariman et al., 2013).

Regarding fatigue, the nature and intensity of the reported symptoms seems different in primarily fatigued individuals, such as in individuals with insomnia disorder and patients with CFS. While both patient groups often complain about intense fatigue (as measured by the Fatigue Severity Scale (FSS; Krupp et al., 1989), fatigue complaints tend to be more related to cognitive or mental fatigue in insomnia, whereas CFS patients present with more physical complaints or a combination of both physical and mental fatigue. More specifically, CFS patients tend to complain about how performing ordinary daily activities (e.g., grooming, household tasks, taking care of children, …) rapidly exacerbates essentially physical fatigue and increases their desire to rest. Conversely, patients with insomnia often can maintain acceptable levels of physical activities, but also express the desire to mentally disengage from tasks they are performing. Regarding cognitive abilities, insomnia patients are even able to maintain relatively intact performance in comparison to controls (Shekleton et al., 2010). It has been hypothesized that mental fatigue is thus more related to the recruitment of compensatory cognitive effort to sustain normal levels of performance (Orff et al., 2007; Hockey, 2013). Insomniacs regularly erroneously respond to fatigue symptoms by trying to sleep, and when sleeping does not occur, they tend to stay in bed so that “at least they can find some rest.”

Clinical experience is often in line with descriptions of fatigue symptomatology found in the literature with respect to rest propensity: fatigue is referred to as “tiredness or the need to rest” (Freal et al., 1984); “subjective need to rest” (David et al., 1990); “unusual need for rest” (Glaus et al., 1996). Bazelmans et al. (2001) state that “CFS patients often complain that physical exertion produces an increase of complaints, leading to a greater need for rest and more time spent in bed.” Furthermore: “… CFS patients reported more minutes resting on the day of the exercise test” (Bazelmans et al., 2005). Similar descriptions are found in other conditions such as cancer: “For example, patients with cancer have described fatigue mostly as physical sensations, such as decreased performance, weakness, and an increased need for rest, but also in terms of sadness and mental tiredness” (Hägglund et al., 2008) or in chronic heart failure: “In a group of elderly with CFS over 80% reported fatigue, shortness of breath, having difficulties to walk or climb stairs and having to rest during the day” (Hägglund et al., 2008).

Whether fatigue presents as physical or mental, psychological factors such as catastrophizing [as in dysfunctional, negative appraisal and attention to symptoms (Severeijns et al., 2001), this may contribute to amplified symptom experience and increased emotional distress (Sullivan et al., 2001)].

In this context, it has been proposed to rename CFS in Systemic Exertion Intolerance Disease (SEID; The Lancet, 2015), by emphasizing central aspects of the condition, however, reviving the outdated mind-body duality (Sen et al., 2016). Indeed, beside the centuries old dualism between body and mind, the most recent advances in fatigue science, be it in clinical practice or exercise physiology have seen the rise of a central/peripheral dualism, where researchers often overlook that we are one integrated organism, not a brain versus a body (Pattyn, 2009), as recently pointed out in the field of exercise science by Gibson et al. (2018).

These variations of symptoms and presentations also call for a common denominator in the description of fatigue, and point toward a transactional definition, considering perception differences, as a potential way out.

A recent instrument allowing for a clinical evaluation of fatigue based on a transactional approach may be useful in this context. The Brugmann Fatigue Scale (Mairesse et al., 2017) is an 8-item 4-point Likert scale sharing a similar conceptual background with the Epworth Sleepiness Scale (ESS), i.e., assessing the propensity to engage in the appropriate countermeasure (i.e., rest for fatigue), and comprising a mental and physical counterpart (Mairesse et al., 2017). When investigating BFS subscale scores in different patient populations, the scores on different subscales reflect the individualized needs depending on pathology: rest propensity can be differentiated between physical and mental. This may have implications for behavioral treatment strategies for instance: while some would promote exercise to counteract cognitive fatigue or sleepiness symptoms (Petajan et al., 1996), they may be not appropriate for patients who experience higher levels physical fatigue, as this type of fatigue tends to be exacerbated by increased motor activity (Krupp, 2003; Ament and Verkerke, 2009). Again, this approach demonstrates the usefulness of considering a transactional model of fatigue (i.e., tailored to the patient’s experience) rather than trying to cram contradictory findings into a one-size-fits-all definition.

Diagnosis and treatment of fatigue-related conditions remain complex in clinical settings. Aside from identifying a single cause for fatigue symptoms (e.g., as in influenza) and treating the underlying condition, most cases of chronic fatigue conditions require a holistic therapeutic approach combining physical, psychological, and neuropharmacological treatment strategies. As previously mentioned, fatigue is a complex phenomenon, for which a single biomarker is not identified yet. Objective measures of fatigue depend on assessing either kinetic and dynamic aspects or require multidimensional testing for mental and physical fatigue components, for which no consensus exists today. The clinical assessment of fatigue thus primarily relies on symptom intensity evaluation (Mairesse et al., 2017). Complexities in diagnosis, together with psychological, behavioral, and social issues contribute to the pathogenesis of fatigue-related conditions and thwart its treatment. Comorbidities such as depression, anxiety, post-traumatic stress or pre-existing medical conditions further worsen potential treatment outcomes. Psychological components of fatigue-related conditions such as fibromyalgia, insomnia, or chronic pain, may be approached by cognitive-behavioral therapy, and have been shown in some cases (such as in insomnia) to be effective and recommended as first line treatment preventing the need for drug therapy (Clauw, 2014; Nijs et al., 2018). Yet, they remain often underused in clinical practice, as these approaches are time and labor intensive for both patient and clinician alike.

## Toward A New Model of Fatigue: Transactional and Multidisciplinary

There are still vivid debates regarding the characterization of fatigue, which seem to be driven by some sort of dichotomy within a conceptual field. This is expressed in the cognitive field by the resource-depletion versus boredom/task withdrawal debate. In the exercise physiology field, the opposition between the integrative governor and the motivation/ego depletion account. And in clinical settings, distinguishing between physical and mental fatigue, despite the fact that this differentiation may have beneficial effects on treatment strategies.

Since most of our information processing is unconscious, attempting to measure motivation, or the willingness to exert effort, or the perception of effort through questionnaires is sophistic. Indeed, it is not because a process is central that it is conscious. Hence, our conclusion would be that these controversies between different accounts or definitions of fatigue are mainly semantic. Indeed, there is no methodology to date which allows us to distinguish these concepts with direct measurements. The use of a diverse terminology in distinct fields of research further maintains the blurred concepts of mental fatigue, cognitive fatigue, ego-depletion, decisional fatigue, or motivational fatigue. Which indicate a common phenomenon.

Building on existing models and concepts in all the reviewed fields, we present a new model of fatigue, applicable to cognitive science, exercise physiology and clinical practice. This model is mainly based on Hockey’s (2013) motivational control model of executive control, effort, and fatigue; Marcora’s (2008) psychological-motivational model of exercise performance; and Marcora’s (2016) model of psychological mediators of physical activity behavior. Indeed, all these share a common denominator, as well as the clinical scale we discussed before (BFS), namely the perception of effort as a central construct. As emphasized previously, similarly to the transactional model of stress (Folkman and Lazarus, 1985), this notion of perception of effort allows to account for the interindividual variability in fatigue. Furthermore, it is a feature that allows to overcome the cumbersome distinction between mental and physical, this renewed version of dualism.

**FIGURE 1:**
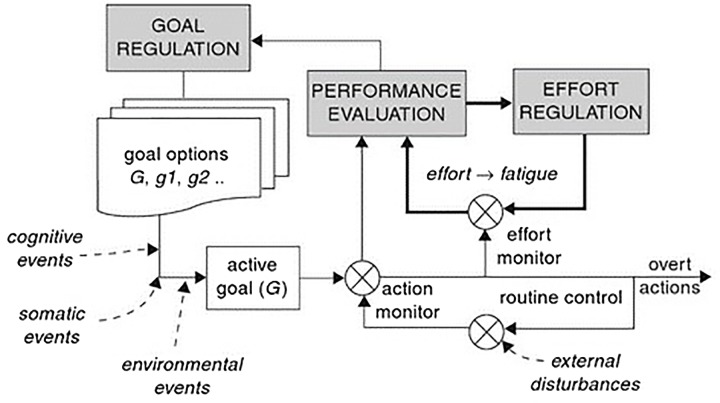
Motivational control model of executive control, effort, and fatigue. This model includes the three executive functions: goal maintenance, monitoring/interruption, and effort regulation. Reproduced with permission from Hockey (2013).

Hockey’s (2013) model (see **Figure 1**) is related to the Motivational Intensity Theory (Brehm and Self, 1989) through the effort regulation system, which is basically the central element with regard to how this model is relevant to our attempt to unify descriptions of fatigue from different fields. Indeed, as pointed out by Hockey in his description of the model, fatigue and effort are both general characteristics of the operation of the whole system, so pinpointing one element or component that would correspond to fatigue is impossible. The effort regulation is crucial, since a sensed need for a greater effort would reflect the same subjective feeling as an increased fatigue. So the frequency and intensity of activation in the effort upregulation might be identified as the key processes in the occurrence of fatigue. A pending question identified by Hockey is whether this activation of the effort regulation loop is the sole mechanism behind a conscious perception of fatigue, or whether it might also arise from the task monitoring process. The concept of “sensed effort,” hence the perception of effort, is the one we are going to build upon in our model.

**FIGURE 2:**
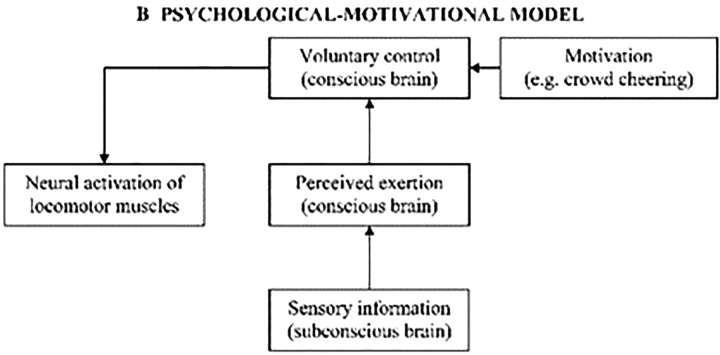
Psychological-motivational model of exercise performance. With this model, Marcora aims at simplifying the pre-existing “Central Governor Model” of exercise tolerance (Gibson and Noakes, 2004) by simplifying the respective roles of conscious and subconscious processing, i.e., by stating that the decision to terminate exercise is a conscious one. Reproduced with permission from Marcora (2008).

Marcora (2008) has attempted for more than a decade to answer the question “when does an athlete give up,” i.e., when does one decide to give up during strenuous exercise. The key component here is the “Voluntary control” part of the process, and as Marcora himself points out, more traditional physiological models of exercise performance have failed to identify the “cardinal exercise stopper,” be it on a systemic metabolic level or a more local muscular level. Hence his argument for a psychobiological model (see **Figure 2**), based on effort-related decision-making. In more practical terms, this implies that the rating of perceived exertion (i.e., the so-called RPE, or Borg scale) actually predicts time to exhaustion, and thus exercise tolerance. Furthermore, Marcora also links his model to the Motivational Intensity Theory, since this theory posits that, for a same level of potential motivation, subjects will decide to stop exercise when a same level of perceived exertion is reached.

The perceived exertion in Marcora’s model is thus similar to the effort monitor in Hockey’s model: the component where the subjective feeling of fatigue arises. The voluntary control in Marcora’s model can thus be related to the effort regulation mechanism in Hockey’s model. These are the key components we will further build upon.

**FIGURE 3:**
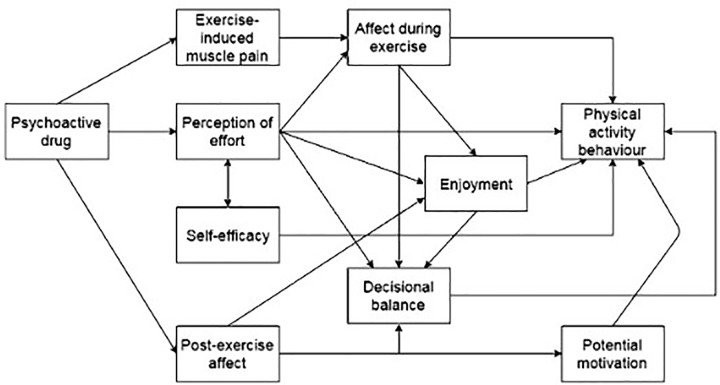
Psychological mediators of physical activity behavior. This theoretical model was illustrating Marcora’s provocative statement about the use of psychoactive drugs to enhance physical activity behavior. Reproduced with permission from Marcora (2016).

The reason we include this model (see **Figure 3**) of Marcora (2016), is because it shows the relative gain of psychological mediators in the paradigm of the volitional control of exercise. Whereas one could argue this shows little added value compared to the previous model, as it merely elaborates on the different psychological constructs, we would argue against this, for it includes the three crucial determinants we have identified so far, being “Perception of effort”; “Decisional balance”; and “Potential motivation.” If we replace the existing output, “Physical activity behavior” with a more generic performance, like “Overt actions,” as in Hockey’s model, we now have building blocks that are applicable to any type of performance alike, be it physical or mental.

**FIGURE 4:**
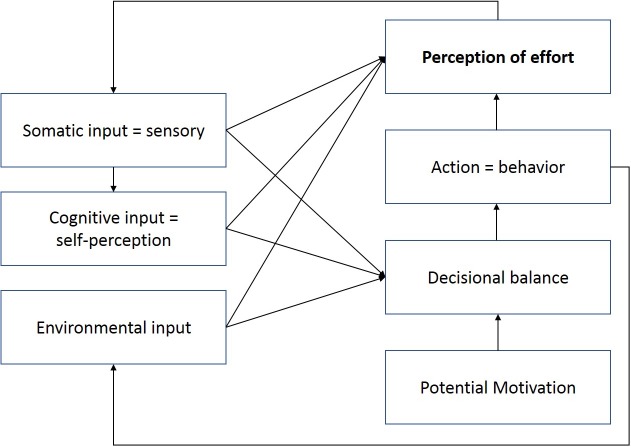
A multidisciplinary transactional model of fatigue and performance. The three key components of fatigue occurrence on the one hand, and performance maintenance on the other hand are the perception of effort; the propensity to exercise effort, which is depicted as the decisional balance; and the potential motivation. Perception of effort is the central component, since it is the one that will generate the subjective feeling of fatigue, which to date is still the most valid way to define the occurrence of fatigue, be it mental or physical. The model is multidisciplinary, as it is valid for all aspects of fatigue, in all types of populations (i.e., clinical or related to supra-normal performance such as elite athletes). It is transactional for it emphasizes the perception of effort as a balance between resources and demands, allowing for situational variations. And it takes into account the regulatory models of performance, by integrating a feedback loop related to the evaluation of performance, which is categorized as an environmental input, for it will depend not only on one’s own perception, but also on the external references (i.e., how I perform compared to others).

As emphasized from previous models and from the Motivational Intensity Theory, we have identified three components that are essential to understand the occurrence of fatigue: the perception of effort; the propensity to exercise effort, which is the product of a decision-making process; and the motivation, which depends from several factors and will influence the propensity to exercise effort (see **Figure 4**). However, we do not agree with Marcora’s distinction of conscious/subconscious processes. Indeed, as illustrated numerously by the seminal work of Kahneman et al. (1982), most of the human decision making is beyond the reach of our consciousness. All our identified critical components (perception of effort, propensity to exercise effort, and potential motivation) are the result of a constant interaction between conscious and subconscious processes, the discussion of which lies beyond the scope of the present paper.

## Conclusion

Fatigue cannot only be reduced to the physical, cognitive, or experiential domain, as demonstrated by our overview. In several disciplines, breakthrough results often stem from crossing boundaries and borrowing and adapting concepts from different fields of expertise. However, too often, related questions are investigated in different domains, duplicating efforts without the profit of multidisciplinarity. Hence our argument in favor of a common transactional model, which would allow to overcome the risk of new scientific results in one area being the emperor’s new clothes in another: fruitlessly reinventing the wheel. This is illustrated by the findings from Angelo Mosso, which are more than a century old. Mosso (in Di Giulio, 2011) defined the laws of muscle exhaustion in his seminal work on fatigue, first published in 1891. He stated, “there is only one fatigue, the nervous […] even muscle fatigue is fundamentally fatigue from exhaustion of the nervous system.” As such, all our novel insights about “mental fatigue” indeed sound like the emperor’s new clothes…

Furthermore, the study of fatigue suffers from a methodological issue related to the study of performance: there is no gold standard to measure it. Whether we measure a cause (in terms of load of physical activity, or in terms of sleep deprivation, or in terms of the duration of an imposed cognitive task) or a consequence (such as a vigilance decrement in terms of performance impairment), or a subjective state, there is no unequivocal signature of fatigue. Despite the recurring notion of depletion of resources associated with the conceptual description of fatigue, as emphasized by Hockey (2013), depletion of energy cannot currently be taken as an explanation for fatigue, except as a metaphor. As Richter et al. (2016) emphasized, a lot of research results question the primacy of the energy conservation principle. The one general conclusion we can draw from the three areas of expertise we inventoried, is that a transactional model fits for all of them, where perceived effort to maintain task goals is the critical determinant of performance, be it physical, mental, or defined as a pathological symptom.

## Author Contributions

NP coordinated the collaboration to write the manuscript. NP, JVC, ED, and OM made significant contributions to the manuscript.

## Conflict of Interest Statement

The authors declare that the research was conducted in the absence of any commercial or financial relationships that could be construed as a potential conflict of interest.
